# A Method for Spatially Registered Microprofilometry Combining Intensity-Height Datasets from Interferometric Sensors

**DOI:** 10.3390/s23084144

**Published:** 2023-04-20

**Authors:** Sara Mazzocato, Claudia Daffara

**Affiliations:** Department of Computer Science, University of Verona, Strada le Grazie 15, 37134 Verona, Italy

**Keywords:** optical profilometry, conoscopic holography, interferometric sensors, spatial registration, art diagnostics

## Abstract

A recognized problem in profilometry applied to artworks is the spatial referencing of the surface topography at micrometer scale due to the lack of references in the height data with respect to the “visually readable” surface. We demonstrate a novel workflow for spatially referenced microprofilometry based on conoscopic holography sensors for scanning in situ heterogeneous artworks. The method combines the raw intensity signal collected by the single-point sensor and the (interferometric) height dataset, which are mutually registered. This dual dataset provides a surface topography registered to the artwork features up to the precision that is given by the acquisition scanning system (mainly, scan step and laser spot). The advantages are: (1) the raw signal map provides additional information about materials texture, e.g., color changes or artist marks, for spatial registration and data fusion tasks; (2) and microtexture information can be reliably processed for precision diagnostic tasks, e.g., surface metrology in specific sub-domains and multi-temporal monitoring. Proof of concept is given with exemplary applications: book heritage, 3D artifacts, surface treatments. The potential of the method is clear for both quantitative surface metrology and qualitative inspection of the morphology, and it is expected to open future applications for microprofilometry in heritage science.

## 1. Introduction

Surface microprofilometry is a well-established technique in the engineering field used for measuring the small-scale topography of precision surfaces that is gaining interest in heritage science thanks to the availability of optical methods [1,2]. Heritage applications are non-standardized and involve peculiar, hand-made artistic objects with a heterogeneous surface, which is subjected to modifications after interactions with the environment or restoration intervention. The artwork surface contains variegate information at sub-millimeter and micrometer scales, from smooth deformations of the support [3] to the texture of the painted layers [4], from artist effects to craquelure or defective patterns [5]. The most commonly employed 3D profilometry techniques include laser triangulation [6], structured light projection [3], and focus variation microscopy [7]. An analysis of the above approaches is given in a recent work by Elkhuizen et al. [5], together with a comparison of the performance of fringe-encoded stereo imaging [8] and 3D digital microscopy on an ancient painting. Microscopy methods provide high accuracy and precision but have a very limited field of view. A surface roughness approach to painting brushstrokes at the micrometer and sub-micrometer scales is given by Mironova et al. [4] using focus variation microscopy and white light interferometry. Reflectance transformation imaging is a more recent full-field technique, based on computational photography, that was shown to sample morphological damage in artworks at sub-millimeter scale (∼0.3 mm) [9]. The joint use of microprofilometry and pointwise spectroscopy is explored in a recent paper by Borg et al. [10]. The massive literature on microprofilometry is based on the use of commercial off-the-shelf instrumentation characterized by high performance at the cost of a non-versatile setup. The main limitations concern the portability, the small field of view, and the sample size, which do not allow the measurement of immmovable artworks in situ, e.g., a large vertical canvas painting.

Among the optical microprofilometry techniques, we recognize conoscopic holography [11] as an optimal candidate that fits the requirements of artwork diagnostics as it enables portable, robust, and versatile measurements [12]. Conoscopic range sensors are efficiently used in precision engineering [13], as well as in medical [14] and forensic [15] applications. Early works in heritage science were done by Fontana et al. [16] on paintings and statues, underscoring the potential for further studies.

Our group is dedicating strong research efforts in the topic: in ref. [17], a prototype with a versatile setup suitable for out-of-laboratory measurements was customized, showing that scanning profilometry based on multiple conoscopic holography sensors and a multi-step data pipeline provides high-quality surface datasets on heterogeneous (diffusive, highly reflective, or polychrome) artworks; in cross-collaboration with conservators, in ref. [18], a multiscale roughness analysis workflow was validated for monitoring the treatments in silver artworks, while in ref. [19] surface metrology based on ISO standard descriptors was applied to in-process and in situ monitoring of ancient paintings. From the work carried out, it emerged that a key step for a reliable analysis of the surface topography was the accurate spatial registration of the profilometry datasets.

The present work is concerned with the open problem in artwork profilometry regarding the difficulty to spatially register the microsurface dataset due to the lack of references in the height data with respect to the “visually readable” surface. Despite the potential of conoscopic holography-based profilometry in heritage science, the problem of spatial referencing the micro-topography is recognized as a limit of the technique, especially in monitoring diagnostic tasks [20].

We do not focus on registering methods involving data processing, for which specific literature is available. The issue is different from that of precision inspection in engineering, where model-based registration techniques are efficiently used [21] to align the conoscopic measurement with free-form surfaces. The conoscopic probe is also coupled to a 3-axis machining center [13] for quality control of mechanical components. The issue is also different from that of registering point clouds into a common coordination system, as in multi-sensor (e.g., fringe-conoscopic) profilometry [22], where the same 3D features are scanned or a calibration target is used. Here we will explore a solution that exploits the ability of the conoscopic holography sensors to record intensity data (i.e., the backscattered signal of the laser diode) in addition to the range information. An algorithmic framework for 3D registration using range and intensity information was early proposed in computer vision [23]. In engineering, a recent paper proposes a topography stitching algorithm based on reflectance and multimap from focus variation microscopy and white light interferometry [24]. While the approach is natural with non-coherent optical systems that capture color or intensity data, it has not been exploited in the case of conoscopic holography systems.

In this work, specifically, we will introduce a new acquisition workflow for the laser scanning conoscopic holography profilometer that goes beyond the usual framework and responds to the need for spatial referencing the micro-topography for in situ art diagnostics.

In the case of works of art, the surface is first and foremost something that is seen. Thus, beside the quantitative inspection of the microsurface asperities retrieved by the interferometric data, we will demonstrate the use of the raw intensity values collected by the conoscopic holography sensor in order to obtain meaningful information from the surfaces. The joint exploration of these two spatially registered datasets proves to be very useful for cultural heritage applications: on the one hand, the surface heights map allows for quantitative measurement of surface topography; on the other hand, the raw intensity map contains some information about the texture materials, e.g., pigments and artist signs.

## 2. Materials and Methods

**Figure 1:**
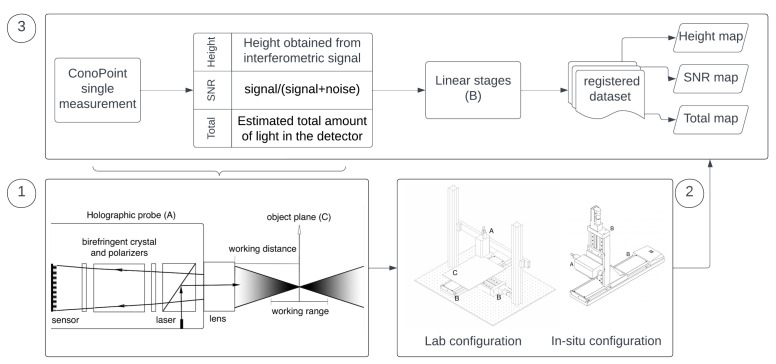
Scheme of the working principle of the microprofilometer. Block 1: Conoscopic holography probe concept and setup. Block 2: Scanning system setups. Block 3: Datasets: single point measurement acquired by the probe and maps acquired through the scanning system.

The sketch in Figure 1 exemplifies the idea behind our prototype and this work, depicting hardware and data modules. Below, we firstly describe the optical scanning microprofilometer, focusing our attention on the performance analysis of the conoscopic holography probe. Secondly, we detail the multiple dataset acquisition and the novel microprofilometry data pipeline.

### 2.1. The Optical Scanner Microprofilometer

The microprofilometer is a customized prototype based on conoscopic holography depth sensors combined with a scanning system of linear stages that are orthogonally mounted [17]. This system allows high resolution and accuracy both in the depth (order of sub- micrometers) and in lateral (order of micrometers) directions, as well as the ability to scan macroscopic regions (order of tens of centimeters). A comprehensive description of the prototype and its applications on artworks can be found in literature [18,19,25].

**Figure 2:**
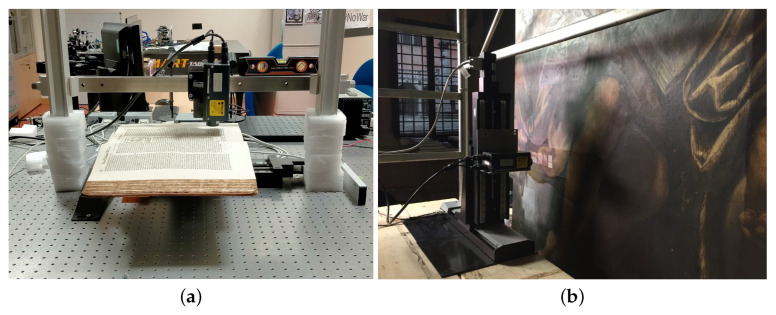
Microprofilometer applications on genuine case studies. (**a**) Laboratory configuration: microprofilometer on the optical table while scanning an ancient book. (**b**) In situ configuration: microprofilometer scanning an ancient large canvas painting by Tintoretto in the Church of the Frari in Venice, further explored by the authors in [19].

The hardware modules are depicted in the scheme in Figure 1 (block 1 and block 2). The holographic probe is based on a laser diode of wavelength 655 nm and provides single-point distance measurements in the line-of-sight direction. The probe is combined with the motion system that enables it to scan areas of 30 cm × 30 cm with an incremental motion of 0.1 μm. The system can acquire objects of different shape and morphology using different lens-probe couplings that tailor the working range to the surface scale. A versatile scanning setup has been designed, tailored to heritage applications: (1) the “Laboratory” configuration is optimized to scan objects on a horizontal plane, with the probe in fixed position and the xy stages moving the sample plate, e.g., for the acquisition of a manuscript; (2) the “in situ” configuration is optimized to scan objects on a vertical plane, with the xy stages moving the probe, e.g., for the acquisition of large paintings. Figure 2 depicts the two microprofilometer setups in the diagnostic campaigns.

The quality of the measurement is assessed by controlling the frequency and the laser power [26], together with the scanning parameters sampling step and velocity of the stages that are set in continuous triggering mode [17].

**Figure 3:**
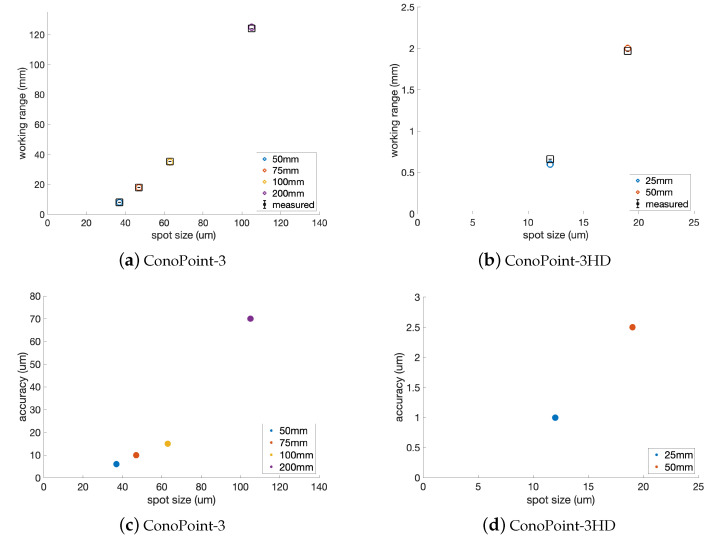
Performance analysis of the different lens-probe coupling. (**a**,**b**) Working range variation versus laser spot variation. The working range has been measured on a flat diffusive standard, the nominal value is also reported. (**c**,**d**) Dependence of depth accuracy on laser spot. Setups: ConoPoint-3 (lenses: 16 mm, 50 mm, 75 mm, 100 mm, 200 mm); ConoPoint-3HD (lenses: 25 mm, 50 mm).

The downside of increasing the working range is the enlargement of the laser spot size. We have carried out a performance analysis for the probes available in the prototype, namely the ConoPoint-3 and ConoPoint-3HD (by Optimet), in the different lens setups designed for multiscale acquisition. In Figure 3, the measured and the nominal values of the working range (i.e., the surface scale) and of the depth accuracy are plotted versus the size of the laser spot (full width at half maximum (FWHM)) at standoff position. As can be seen, scanning objects with a high degree of variation along the z-axis results in a loss of lateral (x-y) resolution (given by the spot size) and depth (z) accuracy. It is worth noting that the spatial resolution is in the order of micrometers and that we obtain a relative depth accuracy (i.e., estimated as the ratio of accuracy to working range) of <0.1% for the ConoPoint-3 (lenses 50 mm, 75 mm, 100 mm, and 200 mm), i.e., when scanning objects in the scale (depth range) of 8 mm to 125 mm, and <0.2% for the ConoPoint-3HD (lenses 25 mm, 50 mm), i.e., when scanning objects in the scale 0.6 mm to 2 mm.

This preliminary performance analysis also characterizes the dual height-intensity dataset, i.e., by putting in relation the spatial resolution of the imaged-based intensity signal (anchored to the laser spot) with the spatial and depth resolution of the height data.

### 2.2. Multiple Dataset Acquisition

The surface acquired by the optical microprofilometer can be regarded as a 2D continuous function of heights sampled by the instrument at discrete points in the selected region of interest (ROI), with the spatial x-y grid given by the scanning step. Following the scheme in Figure 1 (block 3), in addition to the height data obtained from the interferometric signal, the conoscopic holography probe acquires at a single point the signal-to-noise ratio (SNR) and the total intensity signal collected by the sensor (Total). Therefore, each experimental dataset provides a set of three surface maps that are spatially registered with micrometer precision and contain different information. Details are given in Table 1.

By controlling the frequency and the laser power, the SNR and the total values are adjusted at the measurement point in order to evaluate the quality [26].

The novel idea presented in this work is to exploit the intensity dataset Total to detect surface changes such as edges and color changes in heterogeneous objects and to propose the systematic use of this additional information for (1) solving the problem of spatial registration and data fusion in microprofilometry and (2) enabling precision diagnostic tasks that are otherwise not possible.

## 3. Validation Test of the Intensity Signal Total

Working on heterogeneous materials, different absorptions affect the return signal, thus requiring an adjustment of the CCD exposure (laser power or CCD frequency). In order to investigate the feasibility of combining the height and intensity datasets, first we designed a test to assess the repeatability of the Conoprobe total signal in the acquisition of polychrome surfaces. We collected 10,000 static distance measurements at the middle of the probe’s working range and at its extremes, in the so-called far-range and near-range domains (see block 1 in Figure 1). We found that the standard deviations of the total values are nearly constant along the working range and lower than 0.1 %. The signal stability for a fixed probe-lens setting means that reliable information, i.e., non dependent on the scale of the surface (i.e, the height data), is provided by the intensity dataset total.

**Figure 4:**
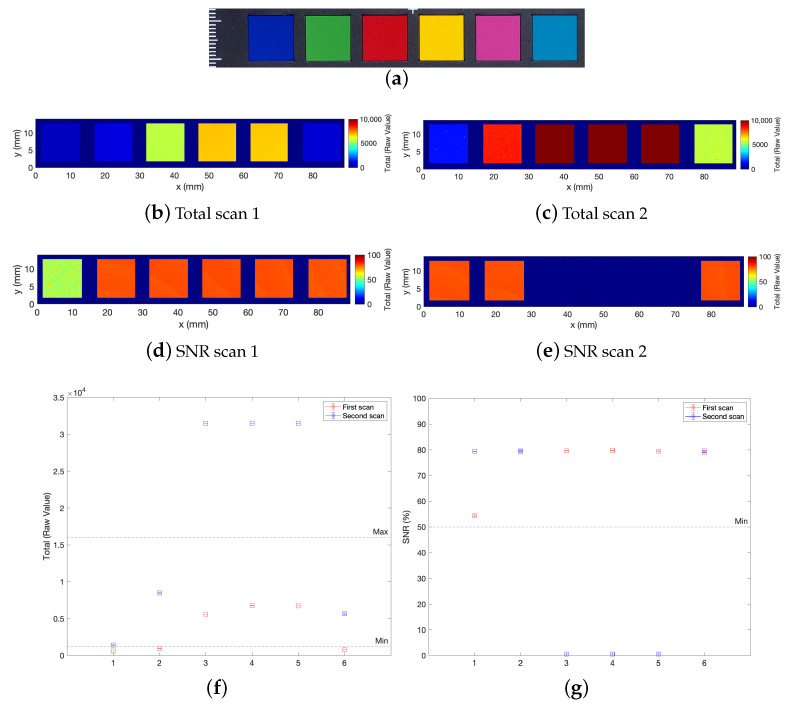
Feasibility test on a colorchecker. (**a**) Visible image of the acquired ROI. (**b**,**c**) Map of the Total values obtained with two optimized power settings. (**d**,**e**) Map of the SNR obtained with two optimized power settings. (**f**,**g**) Value of the Total (left) and of the SNR (right) averaged over the single color patch, with the acceptance threshold indicated; the x-label indicates the column in the colorchecker. Profilometer setup: 50 mm lens, 25 μm scan step, 10 mm/s scan velocity.

Then, in order to test the signal on a polychrome surface, a flat colorchecker was acquired. As mentioned, there is a specific range of total and SNR values in which the distance measurement is considered optimal. Beyond such standard quality indicators, commonly used in conoscopic holography systems [27], adjustment recommendations that take into account the influence of surface roughness have been proposed in precision engineering [29]. A two-session measurement was performed, optimizing the laser power to collect the darker or lighter ROIs. In the first scan, the course power (arbitrary unit) was set at 9 (16 fine power); in the second scan, it was set at 20 (48 fine power). The maps in Figure 4b,c show how the raw intensity total values depend on the color; the SNR is good for almost each ROI (Figure 4d,e). The plots in Figure 4f,g report the mean value of the total and of the SNR averaged over the single color patch and the quality indicators. The results show that even if is often not possible to acquire a heterogeneous polychrome surface in a single scan, a multiple-session measurement allows to effectively acquire both the datasets Total t(x,y) and heights z(x,y).

**Figure 5:**
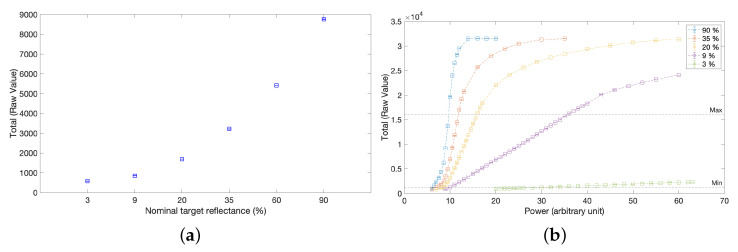
Linearity test on diffusive reflectance standards. (**a**) Variation of the intensity signal Total with respect to the change in target reflectance for a fixed power configuration (course power (arbitrary unit) set at 9, 16 fine power). (**b**) Variation of the intensity signal Total as the laser power varies and as the target reflectance varies.

Eventually, the linearity of the intensity signal total was tested using a set of diffuse reflectance standards (Figure 5). In detail, Figure 5a shows the variation of the signal as the reflectance changes, with the laser set as in the first scan. Furthermore, we studied the linearity of the signal by varying the laser power over the entire available range and by measuring certified standard targets of gradually lower reflectance (Figure 5b).

The tests carried out thus confirm the feasibility of combining the intensity-height datasets in the microprofilometry workflow.

## 4. Proof of Concept Applications

In this section, we demonstrate the joint use of the intensity-height datasets to guide the microprofilometry analysis and obtain more informative results. The additional total signal map is shown to provide meaningful information on heterogeneous surfaces in the field of cultural heritage. Proof of concept is given on three exemplary case studies addressing different tasks in surface profilometry:

- an ancient book, as example of variegate materials on a flat support, in which the Total map enables sub-domain segmentation on surface height data.;
- a painted vase, as example of three-dimensional polychrome archaeological artifact, in which the Total map is used for spatial registration and data fusion;
- the multi-temporal monitoring of a surface texture subject to treatments, in which the total map is crucial to reliably perform surface metrology.

### 4.1. Book Heritage

Profilometry was performed on a 17th-century book from a private collection, at different resolution scales.

**Figure 6:**
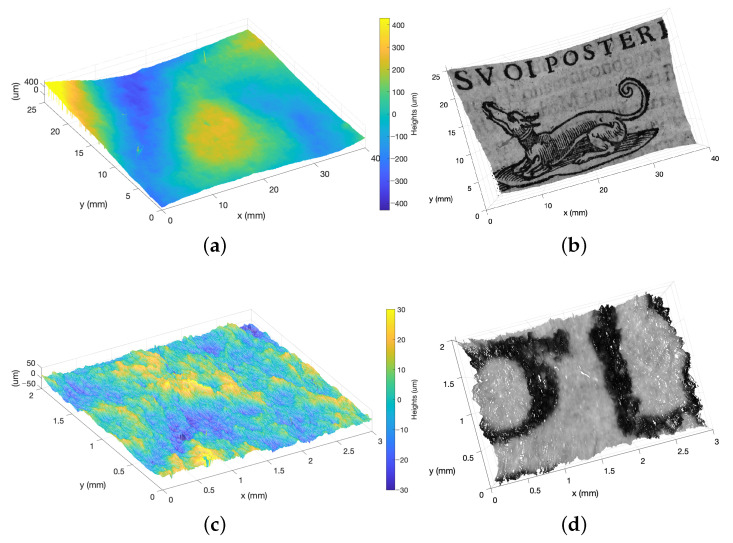
Combining intensity-height datasets from profilometer in book heritage acquisition. (**a**,**c**) Height maps. (**b**,**d**) Total values mapped on the point cloud obtained from the height data. Profilometer setup: (Top) ConoPoint HD, 50 lens, 10 μm scan step. (Bottom) 25 lens, 5 μm scan step. Scan velocity 5 mm/s.

Figure 6a,c show that the microprofilometer effectively acquired the microsurface z(x,y) allowing to capture the deformation (i.e., the waviness) and the roughness of the paper support, but the lack of references in the height data raises difficulties in the interpretation. The use of the raw intensity signal map t(x,y) solves this problem. Figure 6b,d report that, as a result, the Total values mapped on the corresponding surface height map, thus creating a new representation of the surface as intensity total function t(x,y,z). The relevance to turn the surface “visually readable” is evident. In fact, the combination of the intensity-height datasets allows a visual inspection of the surface through the Total function and a quantitative supervised analysis through the surface heights map.

As an application example on book heritage, from the Total function it is possible to segment the map, distinguishing the background (i.e., paper) from the wording (i.e., ink) as specific domains for the computation of the roughness parameters. We found a similar Sq roughness: (9.86±0.04) μm for the paper and (10.72±0.05) μm for the printed areas.

It would not have been possible to perform this task and obtain this result using only the microprofilometer height dataset, as it did not provide enough information for sub-domain segmentation.

### 4.2. Three-Dimensional Polychrome Artifacts

The vase is characterized by painted decorations and surface profilometry carried out in rich polychrome detail.

**Figure 7:**
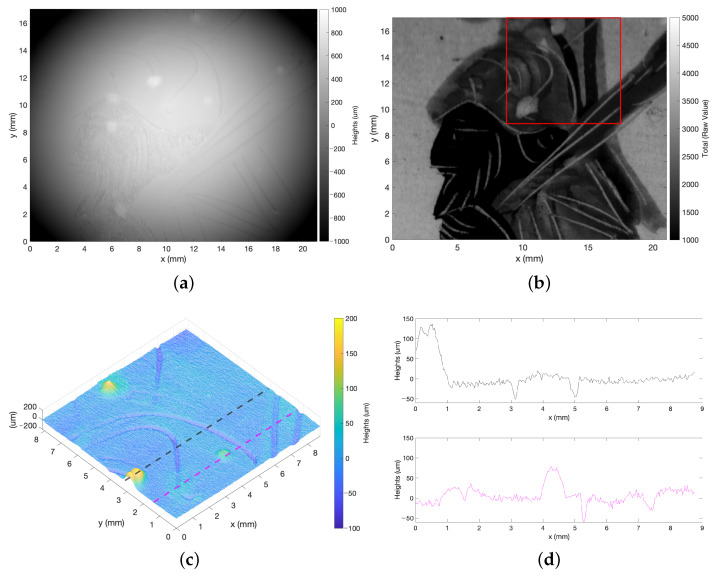
Combining intensity-height datasets in 3D polychrome artifacts: (**a**) Height map obtained by combining two optimized measurements. (**b**) Total values of the first scan mapped on the height map. (**c**) 3D visualization of the roughness texture of the highlighted red ROI. (**d**) Profiles plots exploration with reference to (**c**). Profilometer setup: Conopoint3, 75 lens, 25 μm scan step, 10 mm/s scan velocity.

In order to effectively acquire the heterogeneous ROI, a double scan was performed with different laser power settings and then combined using the SNR dataset [17]. The first scan (laser power: 9 arbitrary unit) allowed the acquisition lighter colors; the second scan (laser power: 23 arbitrary unit) allowed the acquisition of darker areas. The reconstructed surface shown in Figure 7a was obtained by considering the measurement with the best SNR for each point. However, surface analysis is complicated by the lack of references in the height data. This drawback is overcome by exploiting the spatially registered intensity-height datasets acquired by the profilometer.

As an application example, we demonstrate how to interactively explore the micrometer height information by visualizing the surface features through the raw intensity signal. Figure 7b shows the total values mapped on the surface and used to supervise the choice of the detail. The roughness texture was isolated by form removal (Figure 7c). The different incisions and color deposits can be appreciated from the related plots (Figure 7d).

**Figure 8:**
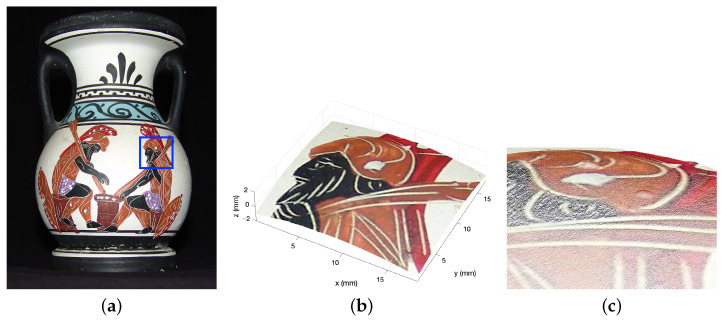
Spatial registered profilometry on 3D polychrome artifacts. (**a**) Visible image of the vase with the analyzed ROI highlighted in blue. (**b**,**c**) Visualization of analyzed surface data as point cloud onto which the visible texture is mapped pointwise.

As a further result, Figure 8 reports the fusing of a visible image of the vase onto the metrological shape provided by microprofilometry. The ROI was extracted from the visible photograph and registered with the Total map shown in Figure 7b, which turns out to be mutually registered with the heights map. The registered image was then mapped onto the point cloud obtained from the height dataset, achieving the final result shown in Figure 8b,c. The polychrome surface texture can be appreciated in the zoomed detail.

This process was only possible through the novel use of the intensity signal map total. Beside nondestructive diagnostics, this application turns to be of potential interest also for heritage documentation and fruition.

### 4.3. Multi-Temporal Monitoring in Murano Glassworks, Venice

This proof-of-concept application was carried out in the framework of the project d3VeRo, involving 3D printing in the art glass sector [30]. This project aims to revive and strengthen the production processes of the Venetian glassmakers, who hold the secrets of one of Italy’s oldest artistic productions. Among the purposes there is the creation of 3D-printed molds suitable for contact with the melt. The analysis of the surface texture was considered of fundamental importance because this information is transferred from the mold to the final artistic glass product. The mold could impart defects to the glass, influencing its visual and tactile appearance.

**Figure 9:**
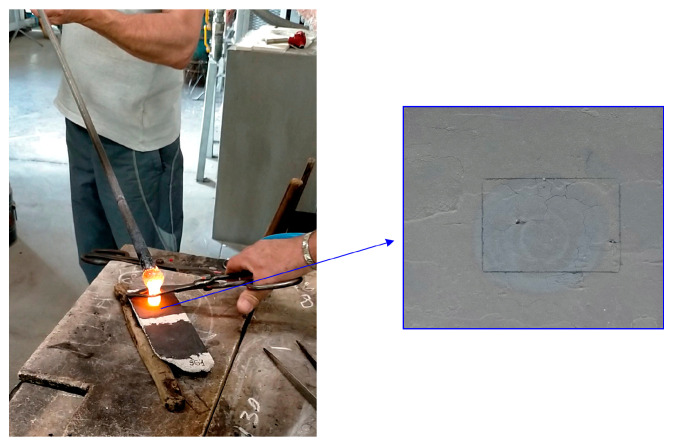
Melt deposition on the surface of the sample in the traditional hand-made process of art glass production. On the right, the spot caused by the melt-mold interaction.

As a demonstration, profilometry is employed for the comparison of the microsurface of the mold before and after the interaction with the melt. Figure 9 shows the moment when the master glassmaker laid the melt on an analyzed sample.

The total maps t(x,y) are used as intensity-based images to find the geometrical transformation that allows for an in-plane registration of the profilometer datasets. The total map after the treatment is registered with the total map before the treatment, taken as reference; the found transformation matrix is then applied to the height dataset in order to achieve two spatially registered surface height maps.

**Figure 10:**
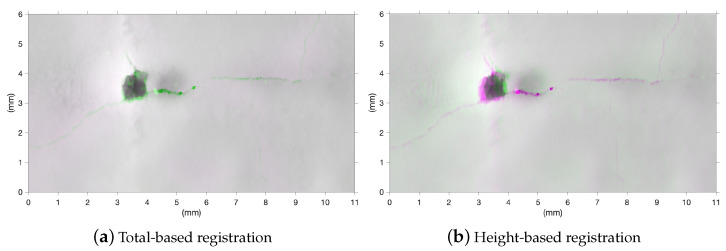
Result of the spatial registration of the surfaces (before/after treatment), shown as false color composite image of the two registered heights maps. (**a**) Registration performed on the Total intensity dataset and then applied to the height dataset. (**b**) Registration performed on the heights dataset. The root mean square error (RMSE) is 18 μm and 26 μm for the Total-based and the height-based registration respectively. The displacement in the crack feature in the height-based results is about 140 μm.

The advantage of performing the registration on the Total maps is evident when we compare the results with those obtained by working on the heights domain. Figure 10 shows an example of the registration of a representative ROI (before/after the treatment) using the standard Matlab algorithm imregister [31]. In this specific case, we found a root mean square error (RMSE) of 18 μm and 26 μm for the total-based and the height-based registration, respectively. In the latter, the loss in accuracy is evident along the cracks, with a displacement of ∼140 μm for the height-based. Clearly, the multi-temporal monitoring of surface metrology parameters is not reliable without an accurate registration of the heights maps.

**Figure 11:**
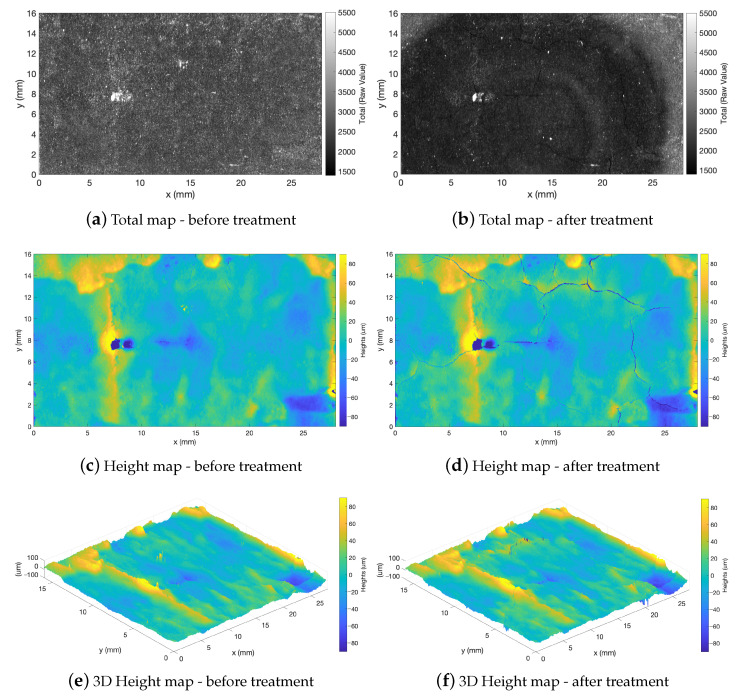
Spatially registered microprofilometry for multi-temporal surface monitoring. The mold was subjected to a treatment in a glasswork and acquired in laboratory in different periods. In-plane registration of pre- and post-treatment surface datasets was obtained by image registration on the intensity Total maps. Profilometer seup: ConoPoint HD, 50 lens, 10 μm scan step, 5 mm/s scan velocity.

The example in Figure 11 shows the temporal evolution of the surface of a mold sample with coating before and after the melt deposition (shown in Figure 9). From the registered heights maps, the formation of cracks due to thermal shock is evident. The joint exploration of the intensity-height datasets provides additional information. The total map after the process in Figure 11b highlights, with a micrometer resolution, the real area of interaction with the melt. This otherwise lost information allows to segment the surface for monitoring the specific ROI. Excluding the cracks from the analysis, we found that the Root Mean Square (RMS) roughness (Sq) varies from (22.12±0.03)μm to (23.47±0.02)μm, the skewness (Ssk) shifts from 0.9 to 0.5, and the kurtosis (Sku) increases from 5.6 to 14.0. This means that the bulk of material is below the mean plane, and the surface exhibits an increase in inordinately high peaks/deep valleys.

It would not have been possible to perform a precision diagnostic without the novel use of the intensity-height datasets. Performing surface metrology on the entire definition area without supervision would have led to a sampling bias and subsequent error in the surface estimators. To clarify, for the considered example, Table 2 reports the supervised and unsupervised computation of the surface amplitude parameters with the systematic errors, which, as can be noticed, are significant.

## 5. Conclusions

We demonstrated a novel workflow for spatially registered microprofilometry of artworks by combining intensity-height datasets from conoscopic holography sensors. The method exploits the raw intensity total signal collected by the interferometric sensor (i.e., the backscattered signal of the laser diode), which is registered pointwise to the interferometric height data and is shown to provide additional image-based information about materials texture (e.g., color changes and artist marks).

The validation tests performed on reference targets proved that the intensity signal provides reliable information across the depth range of the sensor for the different probe-lens settings.

Proof of concept of the methodology was given on the basis of three exemplary applications, addressing tasks in surface profilometry that demonstrate the novel use of the dual intensity-height dataset and its potential in the heritage field.

- The first demonstration on book heritage (variegate materials on a flat support) showed that the intensity total map enabled sub-domain segmentation on surface height data, otherwise not possible.
  The method allowed the computation of roughness parameters on local features such as the paper support and the ink text.
- The second demonstration on a polychrome vase showed that the dual intensity-height datasets enabled spatial registration and data fusion.
  As the intensity signal turned out to be highly sensitive to a polychrome surface, the micrometric height information was explored by visualizing the surface painted features through the intensity dataset. A visible image was mapped onto the surface data by performing the image-based registration on the intensity domain.
- The third demonstration on the temporal monitoring of surface treatments showed that the intensity total map was crucial for a reliable surface metrology.
  The combined use of intensity-height datasets allowed to define the regions involved in the treatment and to compute the roughness parameters on the correct domain of interest, while an unsupervised analysis would have led to a sampling bias with subsequent errors in the surface estimators.

To conclude, the proposed method for spatially registered profilometry based on dual intensity-height dataser enables the use of the technique for in-process diagnostics of heterogeneous artworks. Moreover, the method turns out to be of great interest for heritage documentation and fruition.

The problem of registering datasets acquired with conoscopic holography-based profilometers was recognized in the literature, and it was the starting point of our research. The importance of changing the paradigm in the investigation of artworks surfaces at small scales is clear both for quantitative surface metrology studies and for qualitative inspection of the morphology. If surface data are properly acquired, namely, registered with accuracy to the artwork features, the microtexture information can be reliably processed for “precision diagnostics” tasks.

## Figures and Tables

**Table 1:** Description of the dataset collected by the microprofilometer [27]. Type definitions from Conoprobe OEM API Reference is also given [28].

Surface Map	Description	Reference
z(x,y) Surface heights	Interferometric data reporting the distance between the object and the sensor.	TMeasurement.Distance
snr(x,y) Signal-to-noise ratio	Dataset with the signal quality of each measured point. An optimal measurement requires an SNR value greater than 50%.	TMeasurement.Snr
t(x,y) Total	Energy collected by the detector (raw intensity signal in arbitrary unit). Optimal values for Total are between 1200 and 16,000 counts (900 to 18,000 in extreme cases).	TMeasurement.Total

**Table 2:** Surface parameters of the mold sample before and after the treatment: computation in the corrected domain (supervised by the intensity map); errors in the parameters caused by the bias in the sampling.

	Sq (μm)	Ssk	Sku
Before treatment	22.12±0.03	0.909±0.004	5.649±0.007
After treatment (Supervised)	23.47±0.02	0.473±0.004	14.018±0.007
After treatment (Unsupervised)	29.37±0.03	−2.529±0.004	42.989±0.007
Estimation error (%)	25.13	634.656	206.668

## Data Availability

The datasets generated and/or analysed during the current study are available from the authors on reasonable request.

## Abbreviations

The following abbreviations are used in this manuscript:

ROI	region of interest
SNR	signal-to-noise ratio
RMS	root mean square
